# Role of melatonin combined with exercise as a switch-like regulator for circadian behavior in advanced osteoarthritic knee

**DOI:** 10.18632/oncotarget.19276

**Published:** 2017-07-16

**Authors:** Yunkyung Hong, Hyunsoo Kim, Seunghoon Lee, Yunho Jin, Jeonghyun Choi, Sang-Rae Lee, Kyu-Tae Chang, Yonggeun Hong

**Affiliations:** ^1^ Department of Physical Therapy, College of Biomedical Science & Engineering, Inje University, Gimhae, Korea; ^2^ Department of Rehabilitation Science, Graduate School of Inje University, Gimhae, Korea; ^3^ Biohealth Products Research Center (BPRC), Inje University, Gimhae, Korea; ^4^ Ubiquitous Healthcare & Anti-aging Research Center (u-HARC), Inje University, Gimhae, Korea; ^5^ National Primate Research Center (NPRC), Korea Research Institute of Bioscience and Biotechnology (KRIBB), Ochang, Korea

**Keywords:** osteoarthritis, metabolic homeostasis, melatonin, exercise, circadian clock

## Abstract

Here, we show the role of melatonin combined with or without exercise as a determinant of multicellular behavior in osteoarthritis. We address the relationship between the molecular components governing local circadian clock and changes in the osteoarthritic musculoskeletal axis. Melatonin was injected subcutaneously in animals with advanced knee osteoarthritis (OA) for 4 weeks. Concurrently, moderate treadmill exercise was applied for 30 min/day. Morphometric, histological, and gene/protein-level analyses were performed in the cartilage, synovium, bone, and gastrocnemius muscle. Primary cultured chondrocytes repeatedly exposed to TNF-α were used in an *in vitro* study. The symptoms of OA include gait disturbance, osteophyte formation, and abnormal metabolism of the extracellular matrix (ECM) of the cartilage. Low-level expression of clock genes was accompanied by aberrant changes in cartilage specimens. Nanomolar doses of melatonin restored the expression of clock-controlled genes and corrected the abnormal chondrocyte phenotype. Melatonin combined with or without exercise prevented periarticular muscle damage as well as cartilage degeneration. But prolonged melatonin administration promoted the proteolytic cleavage of RANKL protein in the synovium, leading to severe subchondral bone erosion. These musculoskeletal changes apparently occurred *via* the regulation of molecular clock components, suggesting a role of melatonin as a switch-like regulator for the OA phenotype.

## INTRODUCTION

The socioeconomic burdens associated with the care of OA patients is predicted to increase as societies age. Ongoing research seeks to eliminate aberrant chondrocyte behavior, such as hypertrophic differentiation [1], mineralization [2], and calcification [3], but no curative treatment has yet been found. Time-dependent treatment (chronotherapy) has been suggested to be useful in the management of OA patients, in whom daily rhythms of symptoms (i.e., pain, stiffness, and manual dexterity) are evident [4]. Rhythmic gene expression (∼3.9% of all expressed genes) controlled by the circadian clock is apparent in cartilage specimens [5, 6]. The core clock machinery is composed of genes including *Clock*, *Bmal1*, *Per*, and *Cry* [7]. The CLOCK and BMAL1 proteins dimerize to activate transcription of the *Per* and *Cry* genes, reciprocal expression of which contributes to rhythmic regulation of tissue physiology. The transcription factor *Bmal1* is a well-known pacemaker expressed in many tissues; cartilage-selective *Bmal1* deletion triggered progressive chondral lesions [6]. Tissue-specific clocks are entrained by several cues (i.e., photic stimulation, feeding, and exercise) [8], but both aging and exposure to pro-inflammatory cytokines induce loss of rhythm.

The pineal hormone melatonin (N-acetyl-5-methoxytryptamine) is a potent circadian synchronizer response to photoperiod in the mammals [9]. Melatonin has anti-oxidant and anti-inflammatory properties [10, 11], even though it has long been recognized as a disease-promoting agent in rheumatoid arthritic conditions [12]. However, the pharmacological concentrations (μM to mM) can stimulate not only generation of reactive oxygen species (ROS) but also cytokine production [13]. Our preliminary studies confirmed that moderate-intensity exercise can effectively inhibit the phenotypic changes due to prolonged melatonin treatment [14]. Mild-to-moderate exercise suppresses inflammatory processes [15, 16] by generating biomechanical stimuli mediated by articular compression [17]. Although circadian regulation of the major genes in the musculoskeletal system is considered to be useful for medication, little information is available about the effect of intervention on the regulation of clock mechanism. Here, we assessed the role of melatonin combined with or without exercise as a determinant of clock-controlled behaviors. Moreover, an inflammatory microenvironment in the joint can cause synovitis [18], bone erosion [19], and perimetric muscular weakness, contributing to functional impediment [20]. Thus, we performed a holistic approach to correct the changes occurred in the OA musculoskeletal axis.

## RESULTS

### Melatonin with or without exercise inhibited the metabolic shift toward catabolism in advanced osteoarthritic cartilage

**Figure 1:**
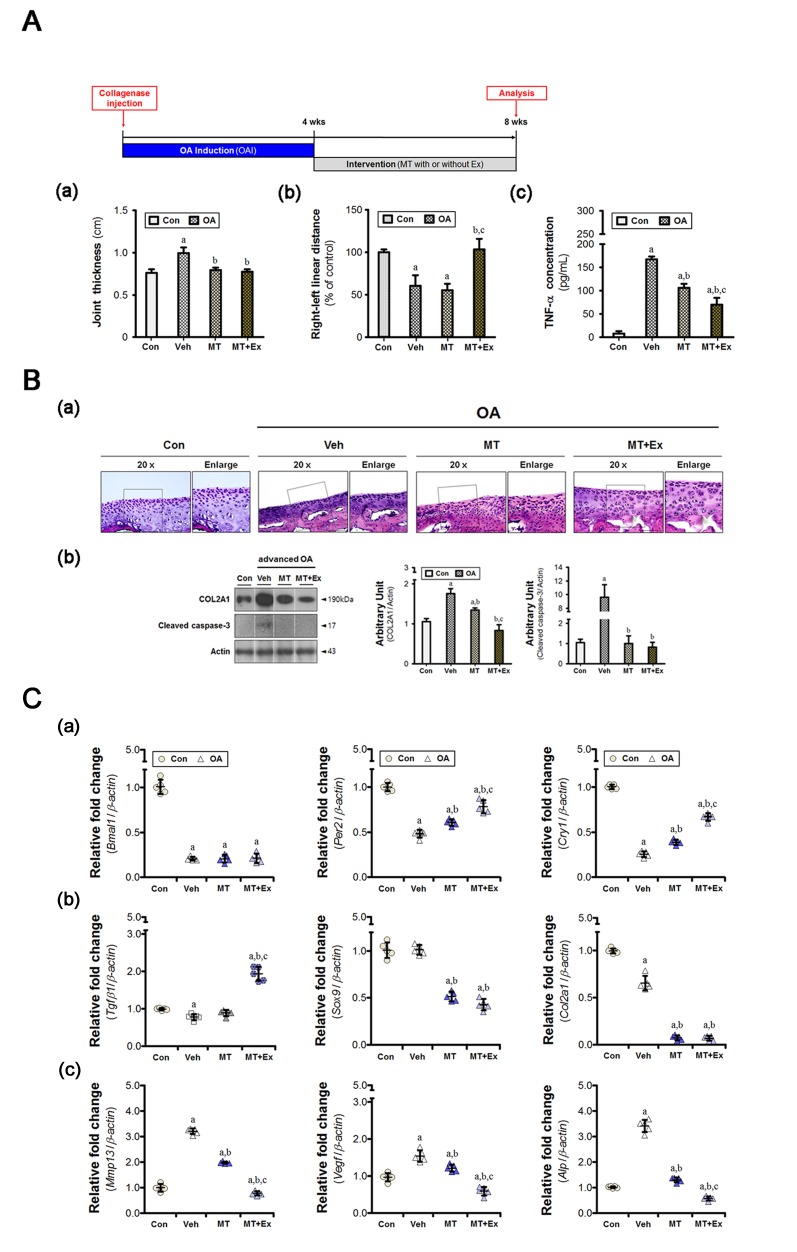
Melatonin combined with exercise restores metabolic balance in osteoarthritic cartilage **A.** Collagenase was injected to induce moderate-to-severe knee OA in animals, and the intervention was applied for 4 weeks. (a) Anteroposterior knee thickness was assessed with calipers in anesthetized rats (*n* = 8); the value increased in cases of advanced OA. (b) The right-to-left linear distances (*n* = 24 for each condition) were normalized with the value of the control rats. (c) The concentration of TNF-α present in serum was reduced by the intervention, the degree of which was greater in combination with exercise. **B.** (a) Co-treatment blocked histological deterioration in the tibial cartilage specimens. Magnification ×40, scale bar = 100 μm. (b) Immunoblot to detect the indicated proteins (COL2A1, caspase-3) showed that abnormal metabolic control occurred in the OA cartilage. **C.** The cartilage specimens were harvested at ZT14. (a) Aberrant expression of genes associated with chondrocyte behavior was accompanied by dysregulation of molecular clock components (*Bmal1*, *Per2*, and *Cry1*). Melatonin with or without exercise maintained the homeostatic balance *via* the suppression of (b) anabolic (*Tgfβ1*, *Sox9*, and *Col2a1*) and (c) catabolic (*Mmp13*, *Vegf*, and *Alp*) factors. PCR data were normalized to *β-actin*. ^a^*P* < 0.05, *vs*. Con, ^b^*P* < 0.05, *vs*. OA+Veh, ^c^*P* < 0.05, *vs*. OA+MT.

The anteroposterior thickness of the knee increased in animal with advanced OA (Figure 1A, a). Also, the right-to-left walking distance decreased, indicating that the ability to support the damaged knee was compromised (Figure 1A, b). These changes were accompanied by an increase in the serum TNF-α level (Figure 1A, c), which was slightly lower in the melatonin-treated group, but higher than in the control. The serum TNF-α level was reduced by exercise. The knee thickness returned to near-normal, similar to that of controls, both experimental groups (Figure 1A, a). However, melatonin treatment alone did not ameliorate the gait disturbance, indicating that the functional impediment persisted (Figure 1A, b). The extent of loading tolerated by the injured limb improved when melatonin treatment and exercise were combined.

Chondrocytes were homogeneously distributed throughout the cartilage in hematoxylin-eosin (H&E)-stained images of the Con group (Figure 1B, a), but images from the vehicle-treated group exhibited the typical histological features of OA (i.e., cleft formation, surface irregularities, and tidemark duplication). Type II collagen protein (COL2A1) was overexpressed in OA cartilage, indicative of a homeostatic imbalance in the matrix (Figure 1B, b). Caspase-3 cleavage was also evident. Four weeks of melatonin treatment partially, but not completely, reduced cartilage damage. Furthermore, the degree of inhibition was greater when melatonin treatment was combined with exercise. Aberrant behaviors of chondrocytes were also observed by real-time qPCR. OA cartilage shows abnormal upregulation of catabolic factors, such as *Mmp13*, *Vegf*, and *Alp* (Figure 1C, c). However, the expression of some anabolic factors (e.g., *Tgfβ1*, *Sox9*, and *Col2a1*) was unchanged or suppressed (Figure 1C, b). These pathological changes were accompanied by a marked reduction in expression of the circadian core clock components (Figure 1C, a). The expression of *Per2* and *Cry1* genes, except *Bmal1* gene, was augmented by melatonin treatment combined with or without exercise, the level of which was higher in the combined group. In particular, combined treatment seemed to maintain the metabolic balance between matrix synthesis and degradation through transcriptional suppression.

### Melatonin determined the cellular phenotype during the period of chondrocyte damage

**Figure 2:**
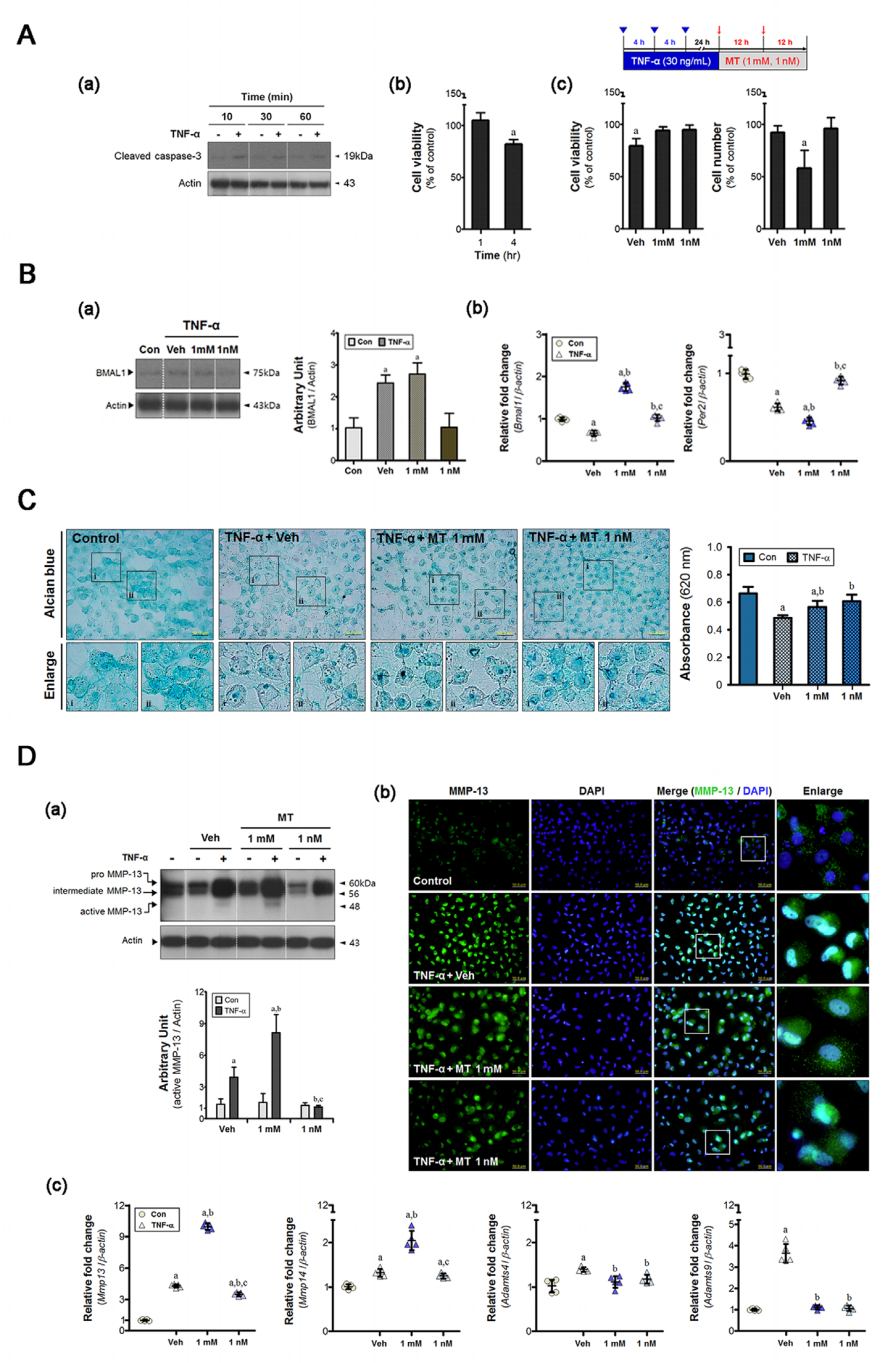
Melatonin determines the cellular phenotype during the period of chondrocyte damage **A.** Primary cultures of chondrocytes (5×10^4^/plate) were synchronized *via* serum shock and treated with recombinant TNF-α (10 ng/mL). (a-b) single dose of TNF-α sustained the damage response for 4 h. (c) Cell viability was reduced by cumulative inflammatory stress (inverted triangle) and the total cell number decreased in the 1 mM melatonin-treated cells. **B.** (a) An inflammatory load abnormally altered the BMAL1 protein level in synchronized cells; millimolar melatonin treatment did not rescue this change. (b) Core clock genes (i.e., *Bmal1*, *Per2*) were differentially expressed in both groups, but remarkable changes were evident upon treatment with 1 nM melatonin. This triggered dysregulation of both the clock and clock-controlled molecules, in turn impairing rhythmic activities. **C.** Alcian blue staining was used to assess the amount of glycosaminoglycans (GAGs). Deposition was higher in the melatonin-treated cells, the degree of which was greater in the 1 nM melatonin-treated group. Magnification ×40, scale bars = 50 μm. **D.** (a) Western blots indicate levels of MMP-13, and its intracellular distribution was determined by (b) immunofluorescence. Nanomolar melatonin treatment inhibited not only proteolytic processing of MMP-13 but also its nuclear localization. Magnification ×40, scale bars = 50 μm. (c) The expression of clock-controlled catabolic genes (*Mmp14, Adamts4/9*) was measured by real-time qPCR. Data were normalized to *β-actin*. ^a^*P* < 0.05, *vs*. Con, ^b^*P* < 0.05, *vs*. DMSO-Veh, ^c^*P* < 0.05, *vs*. 1 mM MT.

Primary cultures of chondrocytes were used to assess the effects of melatonin. Caspase-3 cleavage was initiated within 10 min after addition of TNF-α and was sustained for up to 1 h (Figure 2A, a). Cell viability did not change during this time, but was reduced at 4 h (Figure 2A, b). Thus, we applied cumulative stress via TNF-α treatment every 4 h. Melatonin increased proportion of viable cells, but the pharmacological dose used (1 mM) retarded cell growth (Figure 2A, c). Both the vehicle and melatonin at 1 mM affected the machinery of the chondrocyte clock (Figure 2B). Vehicle-treated cells showed not only an inconsistency between *Bmal1* transcription and translation but also a reduction of *Per2* gene expression. *Bmal1* mRNA abundance was consistent with the protein level in 1 mM melatonin-treated cells. This concentration exhibited a reverse pattern between expression of *Bmal1* gene and that of *Per2* gene. But 1 nM melatonin restored the expression level of clock genes.

Pathological features were evident after disruption of clock machinery, including loss of glycosaminoglycans (GAGs) (Figure 2C), MMP-13 activation (Figure 2D, a), and upregulation of gene encoding clock-controlled catabolic factors (i.e., *Mmp14* and *Adamts4/9*) (Figure 2D, c). 1 mM melatonin promoted marked activation of MMPs at both the transcriptional and protein levels (Figure 2D, a). But the expression of *Adamts* genes was unchanged in 1 mM melatonin-treated cells (Figure 2D, c). Deposition of GAGs remained at a decreased level in the 1mM melatonin-treated group, although the value is higher than that of vehicle-treated cells (Figure 2C). These results indicate high dose melatonin might induce more severe degradation of collagen, rather than proteoglycan. However, abnormal behaviors of chondrocyte were almost recovered by 1 nM melatonin treatment.

We then confirmed MMP-13 localization in inflamed chondrocyte (Figure 2D, b). In the fluorescent images, nuclear MMP-13 was 24.7-fold and 6.2-fold higher in the vehicle- and 1 mM melatonin-treated cells than in the control cells, respectively. Cytoplasmic distribution was also increased in two groups, the degree of which was greater in the 1 mM melatonin-treated cells (∼ 14.5-fold of control) than vehicle-treated cells (∼ 6.5-fold). While MMP-13 protein was facilitated translocation to the nucleus in vehicle-treated cells, addition of melatonin to millimolar levels reduced the facilitation of translocation of MMP protein to the nucleus. The level of cytoplasmic protein in cells treated with 1 nM melatonin was lower than that in cells treated with 1 mM melatonin, although the extent of nuclear localization was similar. Thus, we suggest that melatonin dose may be important when seeking to control the chondrocyte phenotype.

### Melatonin combined with exercise maintained bone homeostasis in joints with advanced OA

**Figure 3:**
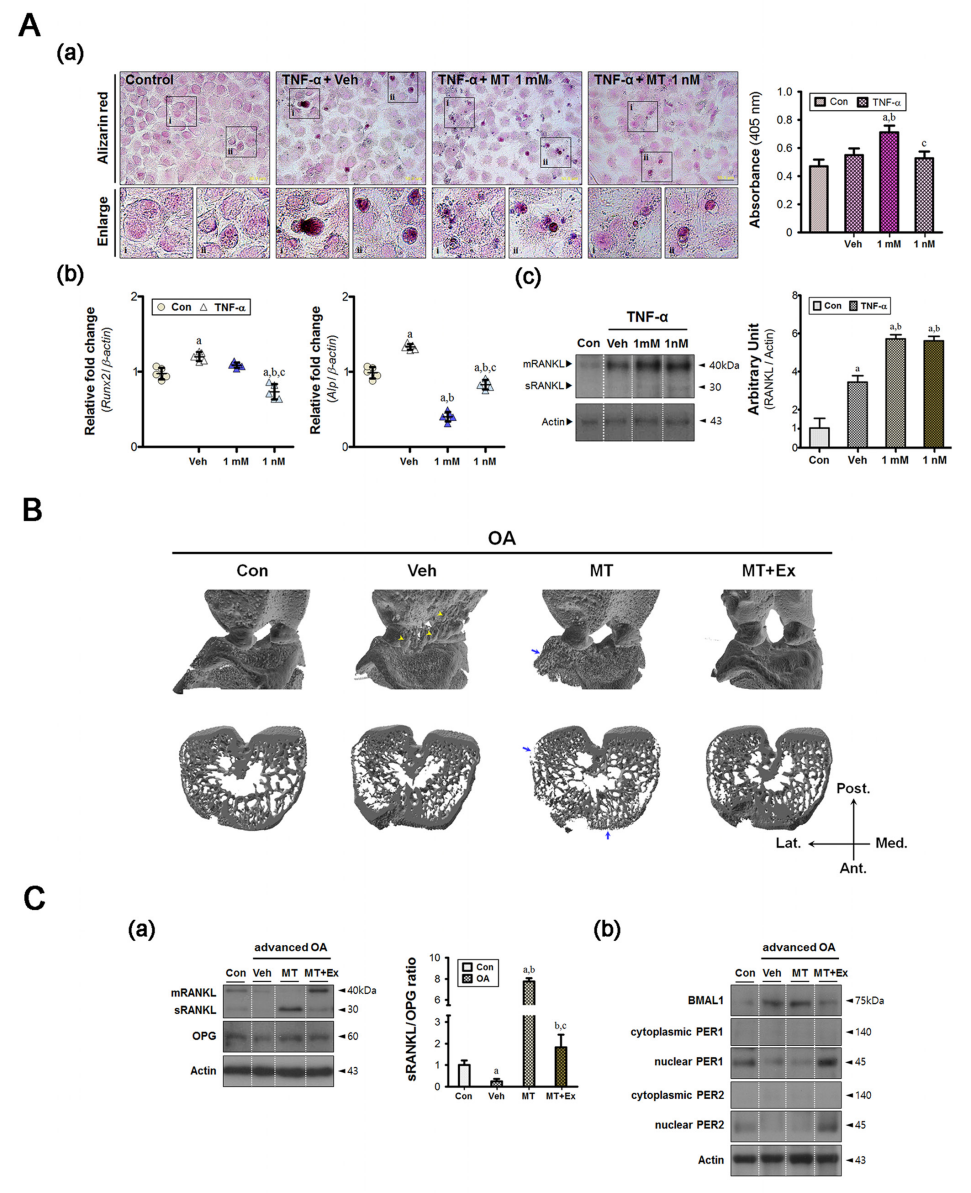
Melatonin combined with exercise maintains bone homeostasis in the advanced OA joint **A.** (a) Ca^2+^ levels were measured by Alizarin red staining; high absorbance was observed in the 1 mM melatonin-treated group. Magnification ×40, scale bars = 50 μm. (b) Repeated inflammatory stimuli increased the expression of hypertrophic markers (*Runx2*, *Alp*) and (c) the level of RANKL. Melatonin treatment suppressed the hypertrophic changes despite higher levels of RANKL protein. ^a^*P* < 0.05, *vs*. Con, ^b^*P* < 0.05, *vs*. DMSO-Veh, ^c^*P* < 0.05, *vs*. 1 mM MT. **B.** Reconstructed micro-CT images showing anterior view (upper panel) and horizontal cross section (lower panel). Yellow arrowhead, calcified tissue; blue arrow, bone erosion. **C.** (a) Immunoblot analysis showed that factors involved in bone remodeling processes were also detected in synovial membranes. Prolonged melatonin administration promoted the conversion of membrane-bound protein (mRANKL) to the soluble form (sRANKL), leading to a marked increase in the sRANKL:OPG ratio in the synovium. (b) The levels of core clock proteins (BMAL1, PER1, and PER2) were abnormally altered in both the vehicle and the melatonin-treated groups, but combined intervention prevented this. ^a^*P* < 0.05, *vs*. Con, ^b^*P* < 0.05, *vs*. OA+Veh, ^c^*P* < 0.05, *vs*. OA+MT.

Although intracellular calcium signaling has an important role in chondrocyte mechano-transduction, it may be associated with pathological features if regulation has failed [21]. From the Alizarin red staining, the quantitative value was higher in the 1 mM melatonin-treated group only (Figure 3A, a). And there were several cell types with stronger signal staining in the vehicle-treated group. Expression of osteoblastic markers (*Runx2* and *Alp*) was enhanced in the vehicle-treated cells (Figure 3A, b), the result of which appeared to be periarticular osteophytes *in vivo* (Figure 3B). The soluble RANKL (sRANKL):OPG ratio decreased in the synovium of vehicle-treated animals (Figure 3C, a), indicating that the intra-articular environment favored bone formation. The inflammatory environment inhibited cytonuclear shuttling of both PER1 and PER2 (Figure 3C, b), which failed to cause degradation of the BMAL1 protein.

In melatonin-treated cells, the level of RANKL protein, an essential mediator of bone resorption via regulation of osteoclastic activity, was consistently high (Figure 3A, c). We found no significant difference in the RANKL protein levels of cells treated with 1 mM and 1 nM melatonin, indicating that the resorptive capacity was preserved under inflammatory conditions. Subchondral bone erosion was marked in melatonin-treated animals (Figure 3B). Quantitative micro-CT revealed that the subchondral bones of the medial tibia became damaged under arthritic conditions, regardless of intervention (Table 1). However, the subchondral trabeculae of the lateral epicondyles became thinned when melatonin treatment was prolonged. Also, the relative bone volume (the BV/TV ratio) decreased in the melatonin-treated group. Microfractures with thin trabeculae were also observed, and the synovial sRANKL:OPG ratio was approximately eight-fold that of the control (Figure 3C, a). The melatonin treatment also altered regulation of the clock proteins, as observed in the vehicle-treated group (Figure 3C, b). The combined intervention corrected these abnormal remodeling processes via recovery of the molecular clock, leading to a reduction in periarticular bony defects.

**Table 1:** Quantitative analysis of osteoarthritic tibiae by micro-CT

	Medial condyle				Lateral condyle			
	Con	Veh	MT	MT+Ex	Con	Veh	MT	MT+Ex
		
BV/TV (%)	34.1±0.8	30.0±1.2^*^	28.7±1.9^*^	28.9±1.6^*^	14.5±1.5	15.1±1.7	12.3±0.7^*^	14.4±1.1
P-value	0.005				0.015			
Tb. Th (μm)	127.9±1.2	112.8±1.5^*^	114.2±5.7^*^	109.4±0.8^*^	100.9±5.0	90.7±2.7	80.4±2.3^*^	100.5±4.0^#^
P-value	0.001				0.011			
Tb. Sp (μm)	233.4±7.2	221.6±6.9^*^	220.2±3.3^*^	232.5±7.1	278.1±9.1	281.3±7.5	275.3±8.5	280.9±7.9
P-value	0.017				0.852			

^*^P<0.05 vs. Con; ^#^P<0.05 vs. MT

### Melatonin treatment with or without exercise were effective for prevention of indirect muscle damage

**Figure 4:**
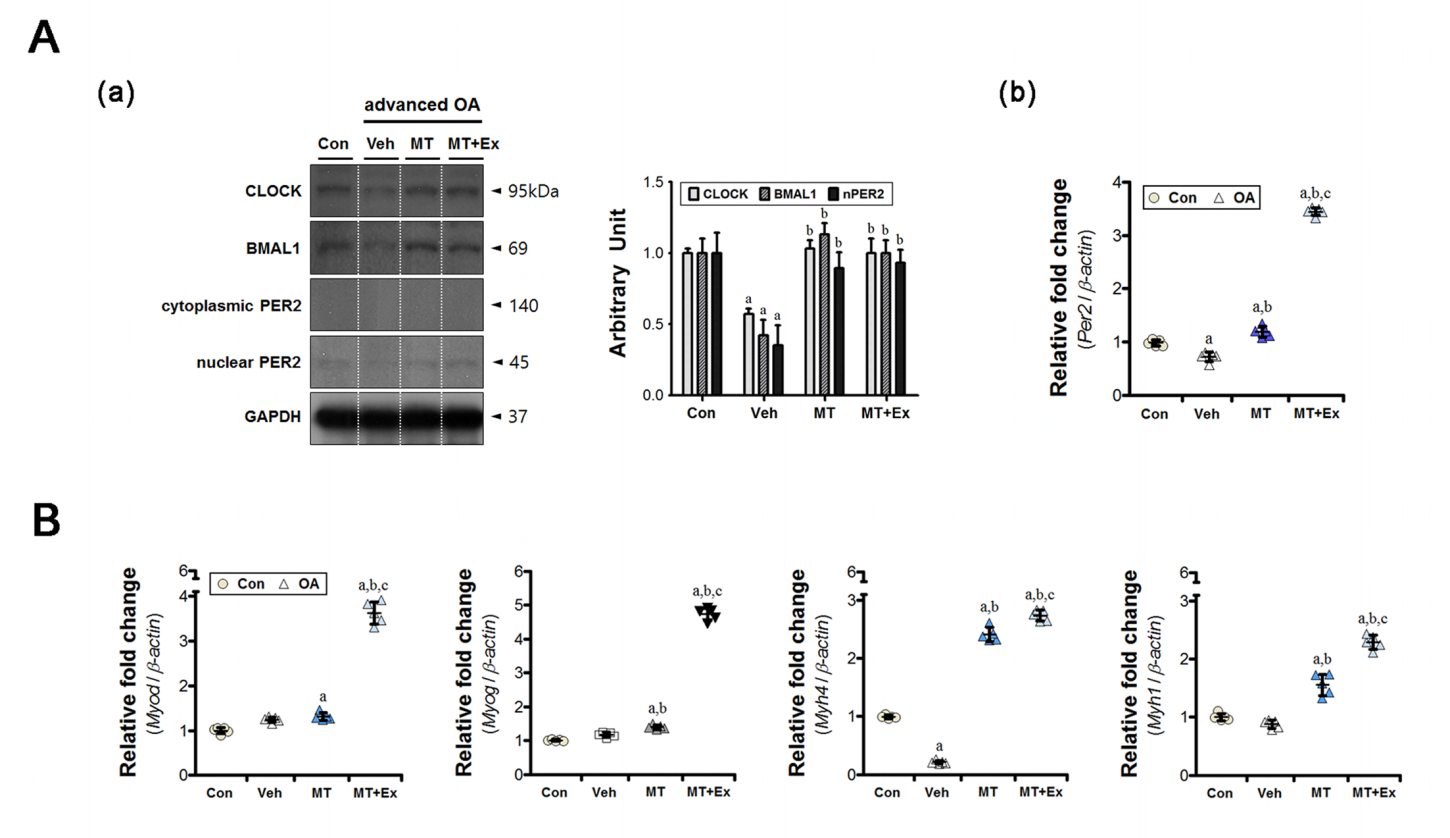
The preventive effects of melatonin with or without exercise on indirect muscular damage **A.** (a) Intramuscular CLOCK, BMAL1, and PER2 levels were analyzed by immunoblotting, (b) and the expression of the clock gene *Per2* was measured by real-time qPCR. The intra-articular inflammatory environment disrupted the expression of core clock proteins in the gastrocnemius, but melatonin and/or exercise restored those levels. **B.** The inflammatory joint altered expression of a gene associated with muscle fiber properties (*Myh4*). Melatonin treatment with or without exercise upregulated the expression of myogenic regulatory factors (*Myod*, *Myog*) and myosin heavy chain isoforms (*Myh1*, *Myh4*). Data were normalized to *β-actin*. ^a^*P* < 0.05, *vs*. Con, ^b^*P* < 0.05, *vs*. OA+Veh, ^c^*P* < 0.05, *vs*. OA+MT.

The ipsilateral medial gastrocnemius initiated the adaptive processes in response to load variation on the injured limb. The muscular clock components were dysregulated at both gene and protein level, similar to that in other joint tissues (Figure 4A). Compared with the control group, the levels of circadian clock proteins (CLOCK, BMAL1, and nuclear PER2) were significantly lower in the vehicle-treated group (Figure 4A, a). Additionally, *Per2* expression was suppressed also (Figure 4A, b). Dysregulation of the molecular clock components decreased the abundance of muscle-specific transcripts markedly. For example, myosin heavy chain IIB protein, encoded by the *Myh4* gene, represents a large proportion of the medial gastrocnemius [22]; its expression was downregulated (Figure 4B). However, the muscle-specific clock-controlled gene *Myh1* was unchanged. Melatonin treatment restored the core clock mechanism, similar to the control; this effect was enhanced when exercise was co-applied (Figure 4A). In particular, the expression of *Per2* was 4-fold higher in the melatonin combined with exercise group. A similar trend was observed with the myogenic factors *Myod* and *Myog* (Figure 4B).

Based on these findings, we suggest that melatonin combined with exercise could efficiently prevent phenotypic alterations in the moderately-to-severe OA musculoskeletal axis, the mechanism(s) of which may be mediated by regulation of circadian clock molecules in joint tissues.

## DISCUSSION

Previously, we reported that circulating TNF-α increased ∼5.5-fold in rats with early ongoing OA [14]. Its level was higher in the present study than the previous result because of elapsed time, equivalent to 8 weeks, which leads to the more advanced OA-like phenotype [23]. This is important because the interventional effect depends on the status at the starting point [24]. TNF-α in culture medium initiated the damage response, and cumulative stress induced hypertrophic changes in primary cultured cells, similar to the animal results. This was accompanied by reductions in not only *Bmal1* and *Per2* gene expression, suggesting that the molecular clock components in chondrocytes are dysregulated during OA progression.

The circadian rhythm can be synchronized in several ways (e.g., by the light/dark cycle or a rest/activity cycle, by exercise, and by feeding times); the output rhythms affect many physiological and behavioral variables (i.e., melatonin secretion, the sleep/wake cycle, and the core body temperature). Mechanical stimuli (e.g., the daily rest/activity cycle and exercise) are major synchronizers of the musculoskeletal clock. The effects of exercise on melatonin secretion have been extensively reported [25-28]. Thus, we explored the effect of melatonin on the maintenance of musculoskeletal physiology. Rhythmic expression of the canonical clock genes (i.e., *Bmal1*, *Per2*) governing metabolic homeostasis was identified in mouse cartilage [5]. Moreover, a loss of circadian rhythmicity in the *Bmal1*-null mice induces age-dependent arthropathy [29]. There are the defects of autonomous clock machinery in primary synovial cells isolated from rheumatoid arthritis (RA) patients [30]. This altered the synovial responsiveness to inflammatory cytokines during RA pathogenesis, suggesting the importance of the local clock. Furthermore, muscle-specific *Bmal1* expression in Bmal1-null mice recovered the level of voluntary wheel-running activity despite an arrhythmic pattern [31]. In our studies, the BMAL1 level at the same time of day differed in cartilage, synovium, and skeletal muscle. Circadian *Bmal1* expression is regulated by the orphan nuclear receptors RORA and REV-ERB α, the differential activities of which determine the tissue-specific BMAL1 level. *Rev-erb α* expressed rhythmically in most metabolic tissues, whereas *Rora* expression is non-rhythmic [32]. The tissue-dependent BMAL1 level might be induced by the differential *Rora* expression, but further experiments are needed to identify the mechanisms involved.

**Figure 5:**
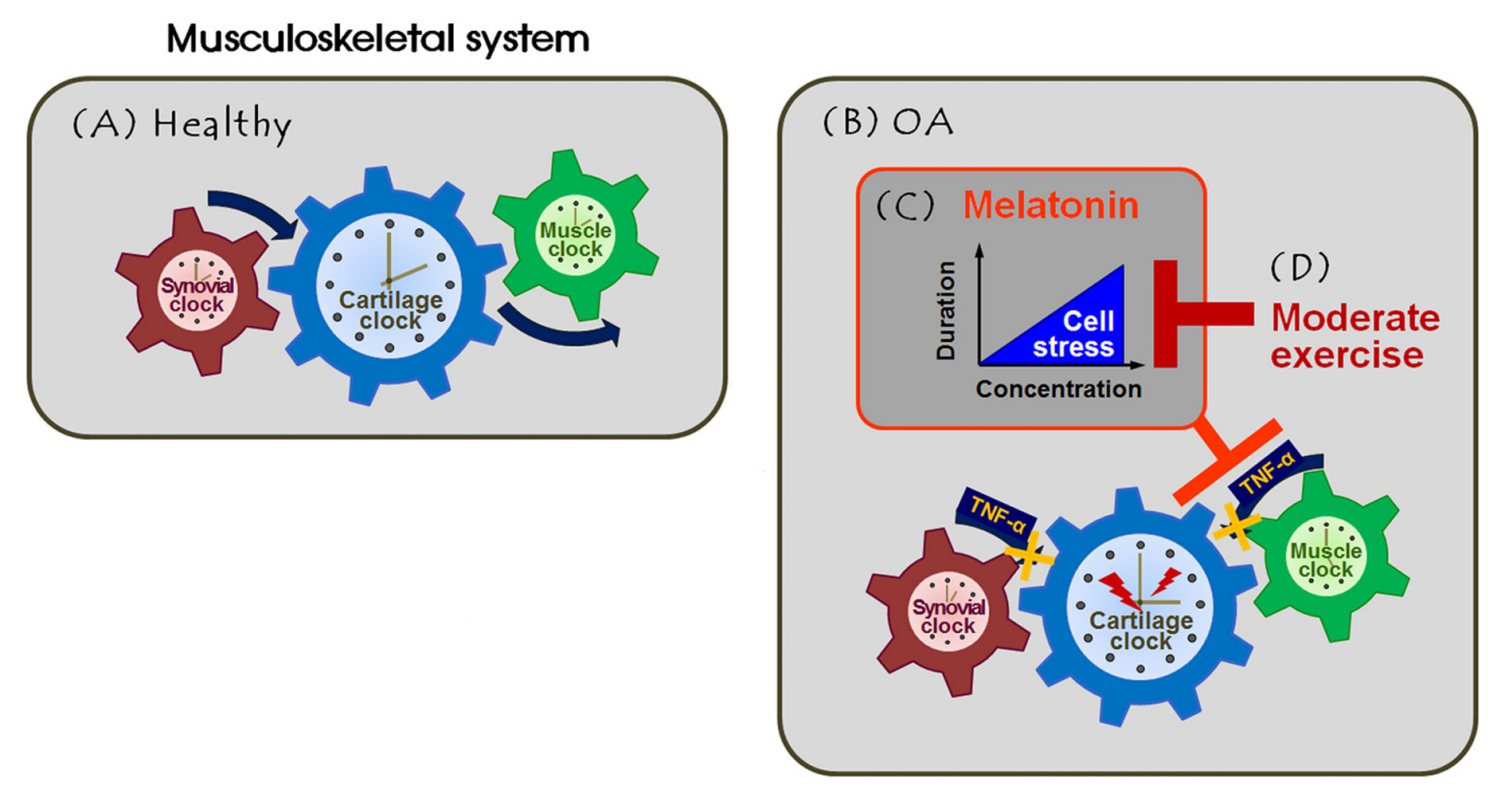
Melatonin combined with exercise rescues the articulomuscular axis injured in knee OA **A.** Local clock systems regulated by the rhythmic expression of genes and proteins are present in healthy joint tissues (cartilage, synovium, and skeletal muscle), co-acting as three cog wheels. This maintains musculoskeletal homeostasis, possibly facilitating coordinated motion. **B.** The arthritic joint amplifies inflammatory signals (e.g., TNF-α), leading to failure of engagement by the tissue-specific clocks. This causes dysregulation of the clock and clock-controlled molecules, which may impair daily activities. **C.** Melatonin at physiological concentrations effectively functioned as both an anti-inflammatory agent and an anti-oxidant in OA joints. However, melatonin damaged cells when given at high doses or long-term. **D.** The deleterious effects of melatonin were inhibited by combining melatonin intervention with moderate exercise. This regime suppressed the development of aberrant axial changes by regulating expression of the clock and clock-controlled proteins of the musculoskeletal unit.

Apart from regulation of *Bmal1*, *Per* gene regulation is critical in terms of joint homeostasis [33]. PER1 and PER2 co-operatively prevent the period length from oscillating to an extent >24 h [34]. However, tissue-specific differences in *Per* gene expression are evident in peripheral organs, the circadian periods of which thus vary. *Per1* rhythmicity and the role played by such variation have been explored only in the synovium [35]. *Per2* gene expression is rhythmic in cartilage [5], the synovium [35], and skeletal muscle [36]. Thus, we analyzed *Per1* and/or *Per2* gene expression in different tissues. The PER proteins are rhythmically expressed in the nuclei of normal synovial cells, but the levels differed (at the time of sacrifice) in cells of the vehicle- and melatonin-treated groups. The *Per* mRNA level peaked at approximately the ZT12 timepoint in the murine limbs, and decreased thereafter [37]. The PER protein should be located in nuclei at ZT14 (the time of sacrifice) because nuclear PER protein inhibits its own transcription. We found that the levels of nuclear PER 1/2 protein were lower in the vehicle- and melatonin-treated groups than in the control group. The BMAL1 protein levels were higher in the former two groups, indicating that circadian phase-shifting might be in play. In fact, melatonin advances the phase of the circadian rhythm in rats with chronic inflammation [38]. RANKL (encoded by *Tnfsf11*) is a clock-controlled gene involved in bone metabolism, and also plays a role in circadian regulation [39, 40]. Thus, the observed phase-shifting may be attributable to melatonin treatment. We applied forced exercise at ZT15; nocturnal rodents commence physical activity at approximately ZT12 [41]. This additional intervention may have reset the local synovial time, allowing bone erosion to recover. Moreover, most RANKL protein was in soluble form in the synovium of the melatonin-treated group, indicating that membrane-bound RANKL may be cleaved by intra-articular MMPs [42]. In turn, this indicates the presence of synovial inflammation in the melatonin-treated group, although melatonin reduced *Mmp13* gene expression in cartilage specimens. However, forced exercise inhibited the synovial inflammatory responses. We observed that the core clock components of skeletal muscle and arthritic joint (the cartilage and synovium) were both dysregulated, perhaps disrupting, in turn, metabolic balance of musculoskeletal tissue. These findings suggest that joint homeostasis is maintained by systemic regulation of peripheral clocks located in cartilage, the synovium, and skeletal muscle (Figure 5). Therefore, we suggest that the anti-arthritic effect of melatonin is determined by several factors, including the dose, treatment duration, combined exercise, and the time of initial intervention.

The COL2A1 protein and *Col2a1* mRNA levels were inconsistent in the vehicle-treated group. Inflammatory signals reduced chondrocyte *Col2a1* mRNA levels and mRNA stability; our results are consistent with those of a previous study [43]. However, regulation at the protein level is more complicated than that at the mRNA level. Type II collagen is abnormally degraded by active MMPs (principally MMP-13) distributed over the entire joint during the early-to-intermediate phase of OA [44], leading to thinning of the surface cartilage. Pro-MMPs activation is inhibited by natural materials (tissue inhibitors of matrix metalloproteinases; TIMPs). Although the cartilage TIMP levels were reduced at the most advanced stages of disease, their concentrations were higher in synovial fluid [44, 45]. Thus, synovial MMPs are barely active in advanced-stage OA tissue, thus increasing the collagen level.

We found that melatonin at a high concentration (1 mM) altered the cellular phenotype compared with that of the vehicle-treated group. Melatonin binds to calmodulin with low affinity, triggering ROS induction [46]. Excessive ROS activate the stress-induced kinase JNK [47], in turn upregulation of *Epas1* expression. HIF-2α (encoded by the *Epas1* gene) increased the cartilage expression levels of matrix catabolic enzymes [48]. The quantitative extent of Alcian blue staining was lower in the vehicle- and 1 mM melatonin-treated cells than in the control cells, indicating that the glycosaminoglycan dynamics may be abnormal in both groups. The expression levels of *Adamts4/9* genes were increased in the vehicle-treated group, reflecting the major roles played by these genes in severe proteoglycan loss. The levels of both MMP-13 protein and the encoding mRNA were increased in 1 mM melatonin-treated cells, although the expression levels of the *Adamts* genes were unchanged. The ADAMTS proteins are involved in proteoglycan breakdown, but membrane-bound MMP-13 and/or the cytoplasmic form degrade small amounts of proteoglycan in arthritic cartilage specimens [49]. High-dose melatonin enhanced cytoplasmic MMP-13 localization, triggering minor proteoglycan degradation. Melatonin at the 5∼10 mg/kg of body weight in animals is known to potentially inhibit MMP activity [50, 51], but the chondrocyte effects that we observed were triggered by nanomolar melatonin, which inhibited both the cytonuclear shift of MMP-13 and protein activity. In contrast, MMP-13 was located predominantly in the nuclei of chondrocytes treated with TNF-α. MMP-13 bears a putative nuclear localization sequence [52], which may explain how MMP-13 becomes transported into the nucleus. MMP-13 was evident in the nuclei of both osteoarthritic chondrocytes and the normal cells [53]. Intranuclear MMP13 promotes the expression of the *Ccn2/Ctgf* genes of chondrocytes, triggering both ECM and cellular proliferation [54]. However, MMPs have been located in cell nuclei under certain pathological conditions (i.e., osteoarthritis, ischemic stroke, and tumors), wherein the MMPs degrade proteins involved in DNA repair and/or transcriptional regulation [55]. Further studies are required to assess whether the intracellular distribution of MMP-13 is associated with disruption of the biological clock.

These insufficient effects of melatonin were blocked by intervention combined with exercise. Although daily strenuous exercise might induce OA [56], low-impact exercise is beneficial [57]. Therefore, moderate exercise could be useful for decreasing the detrimental effects of melatonin at high dose or for the long-term. In fact, the serum TNF-α concentration was reduced to a greater extent with co-treatment. And combined intervention reinforced the myogenic ability as well as the contractile characteristics of muscle fibers through regulation of the local muscular clock. *Myod* is a muscle-specific clock-controlled gene [36], an increase in which may be mediated by recovery of core clock proteins. Changes in musculoskeletal units can alter biomechanical properties [58]. Weight-bearing is a feasible means by which to evaluate OA progression [59]; weight-bearing improved when the two interventions were applied. Thus, we suggest that a combination of melatonin treatment and exercise may exert regenerative effects on the cartilage, synovium, and muscles of patients with advanced OA.

## MATERIALS AND METHODS

### Induction of advanced osteoarthritis and intervention

Male Sprague Dawley rats (8-week-old, *n* = 32) were used. Rats were housed under artificial light-dark conditions at a controlled temperature, and ZT0 was set at 07:00. All rats were provided with water and food *ad libitum*. Experimental procedures were approved by the Ethics Committee for Animal Care and Use at Inje University (approval no. 2010-72). Experimental OA was induced by knee instability via intra-articular collagenase injection in the right knee [14]. At 4 weeks after injection, the degree of gait disturbance was used as a criterion for moderate-to-severe OA. The interventional protocol was described previously [14], and the period was a further 4 weeks (Figure 1A). The rats were given 10 mg/kg melatonin twice daily. Those with moderate-to-severe OA were exercised on a motor-driven treadmill without inclination at 11 m/min, for 30 min/day at ZT15, for 5 consecutive days. Experimental rats were divided into four groups (*n* = 8 for each condition): control (Con), OA with no intervention (OA+Veh), OA with melatonin treatment alone (OA+MT), and OA with melatonin treatment and moderate treadmill exercise (OA+MT+Ex). After sacrifice, specimens were excised at ZT14, snap-frozen in liquid nitrogen, and stored at -80°C.

### Footprint measurements

Footprint analyses were performed as described previously [60]. We measured the linear distance between a print of the right paw and a consecutive print of the left paw (right-left). Each distance was normalized with the mean value of the control rats, represented as % linear distance.

### Serum TNF-α measurement

Serum was stored at -80°C until analysis, then thawed, and run in duplicate. TNF-α levels were quantified using an ELISA kit (eBioscience, San Diego, CA, USA). Immunoassay results were then read with a fluorescence multi-detection reader (Bio-Tek Instruments, Winooski, VT, USA) at 620 and 450 nm. The concentrations were quantitated using the GraphPad PRISM software (ver. 5.0; GraphPad Software, La Jolla, CA, USA). A non-linear regression analysis was used to derive an equation to predict the concentration in unknown samples.

### Histomorphological assessments

Micro-computed tomography (micro-CT) was used to assess OA-induced skeletal deformations, as described previously [14]. After scanning the limbs, we created 3D models of the lesional knee joints. And then we analyzed the quantitative values using manufacture’s software. For histological analyses, tissues were fixed in 4% neutral buffered paraformaldehyde (pH 7.4). Specimens were decalcified with 368 mM EDTA (pH 7.4). Cryosectioning was performed using a cryostat microtome (MICROM International GmbH, Walldorf, Germany). Hematoxylin and eosin (Sigma-Aldrich, St. Louis, MO, USA)-stained images were analyzed using an Olympus DP70 microscope, using a 20× objective and digital camera (Olympus, Tokyo, Japan) connected to a computer.

### Cell culture

Chondrocytes were isolated from the tibial plateau of neonatal rats. At 7 days of culture, the intrinsic clock was reset with serum shock before stimulation with TNF-α. Medium was replaced with DMEM including 50% fetal bovine serum (FBS; Hyclone, Logan, UT, USA) and 1% penicillin/streptomycin (Lonza, Walkersville, MD, USA). After 2 h, the medium was replaced with DMEM containing 1% FBS and antibiotics. Then, recombinant TNF-α (10 ng/mL) was added repeatedly to the medium, three times at 4-h intervals (Figure 2A, c). We then treated twice with melatonin (1mM, 1nM). Melatonin (Sigma-Aldrich) was dissolved in dimethyl sulfoxide (DMSO; Bio Basic Inc., Amherst, NY, USA). The final concentration of DMSO in culture medium did not exceed 0.1%, which caused < 10% cytotoxicity versus untreated control cells.

### Alcian blue and alizarin red staining

We performed Alcian blue staining to compare the amounts of GAGs in the chondrocytes. The dye was extracted with 4 M guanidine-HCl (Sigma-Aldrich), and then measured at 620 nm [14]. Alizarin red, which indicates intracellular Ca^2+^ deposition, was quantified by extraction with 0.5 N HCl and 5% sodium dodecyl sulfate (SDS) and then measured at 405 nm [61].

### Immunofluorescence

Cells were washed, fixed with 4% paraformaldehyde, and incubated in 5% normal horse serum in 0.2% Triton PBS for 1 h. A rabbit polyclonal anti-MMP-13 (Santa Cruz Biotechnology, Santa Cruz, CA, USA) was added to the cells overnight at 4°C. The cells were further incubated with a secondary antibody, CFL 488-conjugated goat anti-rabbit IgG (Santa Cruz Biotechnology), for 2 h. The cells were then washed and mounted with Vectashield mounting medium with DAPI (Vector Laboratories Inc., Burlingame, CA, USA). The stained cells were examined using an Olympus BX51 microscope, using a 40× objective and software (Olympus). To examine the protein distribution, the proportion of MMP-13 localizing with or without DAPI in the cells was quantified using ImageJ ver. 1.48 (NIH, Bethesda, MD, USA).

### Quantitative real-time RT-PCR

RNA was isolated from the specimens using the TRI-reagent (Sigma-Aldrich). RNA (1 μg) was reverse-transcribed using reverse transcriptase (Invitrogen, Carlsbad, CA, USA). The cDNA was amplified with specific primers (Table 2). Each PCR was processed in triplicate. For quantitative real-time PCR, reactions were monitored using a Light Cycler 1.5 (Roche Instrument Center AG, Rotkreuz, Switzerland) with LightCycler FastStart DNA Master SYBR Green I. The *C*_t_ value of β-actin was subtracted from that of the target gene (Δ*C*_t_), and the average Δ*C*_t_ value of the triplicate samples was recorded. The relative gene expression in OA rats versus that in control animals was calculated using the 2^−ΔΔ*C*t^ method.

**Table 2:** Oligonucleotide primers used for PCR

Gene	Primer sequence (5’-3’)	Size (bp)	GenBank Accession No.
			
*Actb*	F: taa aga cct cta tgc caa cac agt	241	NM_031144.2
	R: cac gat gga ggg gcc gga ctc atc		

Anabolic activity-related genes			
*Col2a1*	F: atg aca atc tgg ctc cca aca ctg c	364	[62]
	R: gac cgg ccc tat gtc cac acc gaa t		
*Sox9*	F: aca acg caa gct tct gca ag	111	NM_080403.1
	R: aca ctc tcc aac cac agc ag		
*Tgfβ1*	F: ata cgc ctg agt ggc tgt ct	153	NM_021578.2
	R: tgg gac tga tcc cat tga tt		

Catabolic activity-related genes			
*ALP*	F: cct aga cac aag cac tcc cac ta	138	NM_013059.1
	R: gtc agt cag gtt gtt ccg att c		
*Adamts4*	F: agc ctt taa gca tcc aag ca	153	NM_023959.1
	R: gga ggg ttt agg cct ttc tg		
*Adamts9*	F: gtg gct ctg tac tga agg agc	153	NM_001107877.1
	R: ccg tct cat atg gct tcc tct		
*Mmp13*	F: agg cct tca gaa aag cct tc	226	NM_133530.1
	R: gag ctg ctt gtc cag gtt tc		
*Mmp14*	F: agg gac cct cat agc ttg gt	190	NM_031056.1
	R: tag ggc tca tat gcc caa ag		
*Runx2*	F: gcc agg ttc aac gat ctg ag	201	NM_053470.2
	R: gag gcg gtc aga gaa caa ac		
*Vegf*	F: gcc tca gga cat ggc act at	206	NM_031836.2
	R: gga gga gga gga gcc att ac		
			
Circadian clock genes			
*Bmal1*	F: gtc gaa tga ttg ccg agg aa	101	AB015203
	R: ggg agg cgt act tgt gat gtt c		
*Cry1*	F: ctt cca acg tgg gca tca ac	101	NM_007771.3
	R: ccg aat cac aaa cag acg aga a		
*Per1*	F: ttt gga gag ctg caa cat tcc	101	NM_011065.4
	R: ctg ccc tct gct tgt cat ca		
*Per2*	F: ggc tgt gtc cct ggt ttc tg	101	NM_031678.1
	R: cca caa act tgg cat cac tga		

Muscle-specific genes			
*Myh1*	F: acg gtc gaa gtt gca tcc cta aag	263	NM_001135158.1
	R: cac ctt cgg tct tgg ctg tca c		
*Myh4*	F: agc ctg cct cct tct tca tct gg	229	NM_019325.1
	R: cac ggt tgc ttt cac ata gga ctc		
*Myod*	F: agc ata gtg gag cgc atc t	118	NM_176079.1
	R: gtt ctg cat cgc ttg agg a		
*Myog*	F: tcc cag atg aaa cca tgc cc	111	NM_017115.2
	R: cca ctt aaa agg ccc ctg ct		

### Protein extraction and Western blotting

Specimens were lysed in a buffer supplemented with a protease inhibitor cocktail (Roche) [14]. Concentrations of proteins were measured with the Bradford assay (Bio-Rad Laboratories, Richmond, CA, USA) using a spectrophotometer. Proteins (20 μg) were separated by SDS-PAGE and transferred to a PVDF membrane (Merck Millipore, Billerica, MA, USA). The following primary antibodies were incubated overnight with the blocked membranes: rabbit polyclonal anti-COL2A1, rabbit polyclonal anti-MMP-13 (Santa Cruz Biotechnology), goat polyclonal anti-BMAL1, goat polyclonal anti-CLOCK, rabbit polyclonal anti-OPG, goat polyclonal anti-PER1, goat polyclonal anti-PER2, goat polyclonal anti-RANKL, mouse monoclonal anti-actin, rabbit polyclonal anti-GAPDH, and rabbit monoclonal anti-cleaved caspase-3 (Cell Signaling Technology, Danvers, MA, USA). Membranes were then incubated with the secondary antibody. Finally, specific bands were visualized with chemiluminescent reagents (Pierce Biotechnology, Rockford, IL, USA) and quantified using the ImageJ software.

### Statistical analyses

Data are presented as means ± SD. Significance was determined using one-way analysis of variance with a *post hoc* Tukey test. Differences were considered to be significant at *P* < 0.05. All analyses were performed using the SPSS software (ver. 20.0; IBM, New York, NY, USA).

## Abbreviations

ECM: Extracellular matrix
GAGs: Glycosaminoglycans
H&E: Hematoxylin and Eosin
OA: Osteoarthritis
RA: Rheumatoid arthritis
ROS: Reactive oxygen species
TIMP: Tissue inhibitors of matrix metalloproteinases
